# Characteristics and Performance of PTU-Cu Composite Membrane Fabricated through Simultaneous Complexation and Non-Solvent Induced Phase Separation

**DOI:** 10.3390/polym13111743

**Published:** 2021-05-26

**Authors:** Marianito Tiangson Margarito, Arnel Bas Beltran, Aileen Huelgas-Orbecido

**Affiliations:** 1 Department of Chemical Engineering, De La Salle University, 2401 Taft Avenue, Manila 1004, Philippines; mtmargarito@itdi.dost.gov.ph (M.T.M.); aileen.orbecido@dlsu.edu.ph (A.H.-O.); 2 Materials Science Division, Industrial Technology Development Institute, Department of Science and Technology (DOST), Taguig 1631, Philippines; 3 Center for Engineering and Sustainable Development Research, De La Salle University, 2401 Taft Avenue, Manila 1004, Philippines

**Keywords:** polythiourea, composite membrane, macro-molecule metal complex, phase separation

## Abstract

This study aims to integrate copper (Cu) during membrane formation by a facile simultaneous phase separation process to alleviate biofouling and improve membrane performance. Polythiourea (PTU) polymer synthesized through condensation polymerization of 4,4-oxydianiline and p-phenylene diisothiocyanate in dimethyl sulfoxide was used in the preparation of dope solution. By incorporating different concentrations of cupric acetate in the non-solvent bath, both non-solvent induced phase separation and complexation induced phase separation occur instantaneously. Scanning electron microscopy—energy dispersive X-ray, fourier-transform infrared spectroscopy and time-of-flight secondary ion mass spectroscopy analysis accompanied by color change of the membrane surfaces—confirms the interaction of the polymer with Cu. Interaction of Cu at the interface during membrane formation results in a decrease in contact angle from 2 to 10° and a decrease in surface roughness from 30% to 52% as measured by atomic force microscope analysis. Pure water flux of PTU-Cu membrane increased by a factor of 3 to 17 relative to pristine PTU membrane. Both the pristine PTU and PTU-Cu membrane showed antibacterial characteristics against *E. coli*.

## 1. Introduction

Membrane filtration is becoming one of the most economically viable technologies for the removal of contaminants from water and wastewater [1,2]. However, the gradual increase in transmembrane pressure (TMP) or decline in water flux is still one of the significant factors that increases its overall cost. This is the consequence of membrane fouling formation resulting from prolonged use of the membrane. Biofouling is the most challenging aspect to address due to the formation of a stable biofilm resulting from adhesion and accumulation of biological materials. In many cases this results in irreversible fouling over time which shortens the service life of the membrane [3].

Membrane biofouling is a surface phenomenon and the majority of the foulants, such as extracellular polymeric substances (EPS) and proteins, are hydrophobic in nature. Therefore, it is desirable that the surface of the membrane is hydrophilic to inhibit foulant–membrane adhesion [4]. Furthermore, reducing the surface roughness inhibits fouling by providing less surface area where foulant can adhere. Finally, incorporation of the antibacterial component at the surface also inhibits the attachment and growth of microorganisms.

The integration of the antibacterial component in the membrane structure has been extensively studied to inhibit biofouling. Nanomaterials such as nanosilver [5,6,7], nanocopper [8,9], and nanozinc oxide [10] known to have antibacterial properties are some of the nanomaterials incorporated in the membrane. Given its nano-size, the nature of the material and the required processing prior to incorporation can significantly add up to the cost of the membrane which makes it prohibitive for commercial applications [11]. Out of these materials, Cu has the merit of low cost and antibacterial characteristics resulting from overproduction of reactive oxygen species (ROS), resulting in considerable lipid peroxidation, protein oxidation, and DNA degradation [12,13].

Polythiourea (PTU) and its blends have been extensively studied for application in capacitor dielectrics due to their high dielectric constant, high breakdown field and low dielectric loss [14,15,16]. It has also been applied as an elastomeric coating material [17,18] where some have been commercially used. However, PTU has not been studied extensively for membrane separation until recently.

Villalobos et al. [19] prepared a PTU-metal composite membrane together with polythiosemicarbazide (PTSC). They synthesized the PTU through condensation polymerization of oxydianiline (ODA) and 4,4′-methylenebis(phenyl isothiocyanate). Interestingly, these polymer chelates which contain sulfur in their backbone are able to form polymer–metal complexes as shown by Tomic et al. [20] on PTSC. PTU resin is not available commercially, but was synthesized through condensation polymerization of isothiocyanates and aniline derivatives [21] by microwave assisted polycondensation of thiourea and amine [15] catalyzed by sulfonic acid or by catalyst-free multicomponent polymerization of sulfur, aliphatic diamines, and diisocyanides [22].

The properties of PTU and similar polymers by which they form polymer metal complexes is advantageous for the incorporation of various metals with the desired properties. Its monomeric thiourea unit has been studied and is known to form metal complexes [23,24,25] which is also true of its polymeric form. The addition of metal can have synergistic effects on its performance for applications such as membrane separation and catalysis [26]. Through crosslinking of the polymer, the mechanical property of the membrane is also enhanced at the same time [27,28]. Leaching of the metal is also inhibited, which prolongs its desired functionality.

Since its first discovery by Loeb and Sourirajan [29], the majority of polymeric membranes are prepared via phase separation [30]. This is achieved by changing the conditions surrounding the dope or the polymer-solvent solution [31]. Another technique resulting in phase separation or precipitation of polymer from the dope solution is chemical association such as crosslinking of the polymer through complexation [19] or reaction [31]. Different authors refer to these processes as the complexation induced phase separation (CIPS) and reaction induced phase separation (RIPS) using crosslinkers dissolved in a solvent bath. Unlike some crosslinking processes [32,33] the CIPS and RIPS occur instantaneously through chemical association. By using the same solvent of the dope solution, the polymer-metal complex rapidly precipitates in CIPS/RIPS through crosslinking at the interface. This results in a thin skin layer lacking pores which inhibits the diffusion of the crosslinkers. Completion of phase separation of the support layer through NIPS is achieved in a separate non-solvent bath.

In this work, a Cu infused PTU membrane was prepared through simultaneous CIPS and NIPS. Analysis of the surface morphology and chemistry, cross-section morphology, and Cu distribution were conducted. Membrane properties such as contact angle and surface roughness were also determined to correlate the effect of fabrication process parameters and the incorporation of Cu. The performance of the membrane was also evaluated in terms of pure water flux and the antibacterial property of the surface against *E. coli* as a test organism.

## 2. Materials and Methods

### 2.1. Materials

Polythiourea (PTU) polymer was prepared in the laboratory at room temperature from equimolar quantities of 4,4-oxydianiline (ODA) 98% and p-phenylene diisothiocyanate (PDTC) 97% purity both from Sigma Aldrich (Singapore). Dimethyl sulfoxide (DMSO) > 99.9% purity from RCI Labscan Ltd. (Bangkok, Thailand) was used as solvent for the synthesis of PTU and component of the dope solution for membrane fabrication. Cupric acetate monohydrate (Cu(CH_3_COO)_2_·H_2_O) or Cu(OAc)_2_·H_2_O 98% from LOBA Chemie Pvt. Ltd. (Mumbia, India) dissolved in distilled water was used for the simultaneous NIPS and CIPS reaction. All of the above chemicals were used as received without further treatment.

### 2.2. Polymer Synthesis

PTU polymer was synthesized based on materials used by Ma et al. [21] and the procedure of Villalobos et al. [19] with slight modification. Equimolar quantities of ODA and PDTC reacted in DMSO at room temperature initially with gradual stirring until a viscous solution was formed. The solution was set aside for at least 24 h to further complete the reaction. The dope solution was then precipitated in hot water and dried in an oven at 60 °C for another 24 h.

### 2.3. Membrane Preparation

PTU-Cu Membranes were prepared through simultaneous CIPS and NIPS using 15% PTU in DMSO. In this method, the dope solution was manually cast in a glass plate with a 150-micron (µm) gap casting knife. This was followed by immersion in a distilled water coagulant bath which contained varying concentrations of Cu(OAc)_2_ between 5, 10, and 15 mM. Immersion time in the bath also varied between 5, 10, and 15 s for a total of nine (9) formulations. After immersion in the first bath, the precipitated membrane was then transferred to a distilled water bath for at least 24–48 h to complete the solvent exchange. A pristine PTU membrane was also prepared using distilled water only as the coagulant bath and labelled as PTU. In addition, to investigate the X-ray diffraction (XRD) spectra of different gelation conditions for PTU, a separate PTU polymer sample was prepared by pouring the PTU polymer labelled PTU A in an XRD sample holder and exposed to ambient air conditions for about 30 min until the polymer precipitated and was immersed in distilled water. PTU polymer labelled PTU B was precipitated immediately through immersion in distilled water similar to membrane preparation. The sampling pitch use was 0.02°, voltage of 40 kV and current of 30 mA.

### 2.4. Membrane Characterization

Fourier-transform infrared spectroscopy (FTIR) analysis of the PTU and PTU-Cu membrane was conducted using FTIR Spectrometer Frontier (Perkin Elmer, Waltham, MA, USA) with attenuated reflectance (ATR) accessory using 20 scans within the wavenumber ranging from 4000 to 600 cm^−1^. The FTIR data were further processed and analyzed using KnowItAll Informatics System 2020 by John Wiley Sons, Inc. [34]. The PTU-Cu membrane was also analyzed using a time-of-flight secondary ion mass spectroscopy (TOFSIMS) model TOF.SIMS 5 (Iontof GmbH, Münster, Germany) to determine Cu moieties present at the surface and detected as ions.

The molecular weight (MW) of PTU was determined through the static light scattering (SLS) technique at 90° scattering angle using the DLS model SZ-100 (Horiba, Kyoto, Japan) with an accompanying debye plot automatically generated by the built-in software as presented in the Supplementary Materials. Four (4) solutions of PTU in DMSO with concentrations of 2, 4, 6, and 8 mg/mL were prepared for MW determination.

Scanning electron microscope (SEM) images were taken using Field Emission SEM (FESEM) model helioz nanolab 600i (FEI, Eindhoven, The Netherlands) with energy dispersive X-ray spectroscopy (EDX) (Oxford Instrument X-Max, Abingdon, UK). The membrane samples for cross-section imaging were immersed in liquid nitrogen for about two minutes and fractured. These were sputter coated with a thin layer of gold to prevent sample charging during analysis. The thickness of the skin layer was measured from the cross-section images at 10,000× magnification. The surface morphology and roughness were analyzed in tapping mode using atomic force microscope (AFM) model XE-100 (Park Systems, Suwon, South Korea) with NCHR silicon nitride tip cantilever with a radius of curvature of less than 10 nm. The scan size use for AFM was 5 μm × 5 μm for the determination of root mean square roughness and 500 nm × 500 nm for visualizing the pores and polymer nodules.

Furthermore, the pristine PTU and the PTU-Cu composite membranes immersed for 10 s in the Cu(OAc)_2_ coagulant bath were also subjected to XRD model LabX XRD6000 (Shimadzu, Kyoto, Japan) and analysis with a thermogravimetric analyzer (TGA) model STA 6000 (Perkin Elmer, Waltham, MA, USA).

Contact angle was measured using the sessile drop method with an average of at least five measurements at different locations for each sample.

### 2.5. Membrane Performance

Pure water flux of the membrane was determined using a cross-flow filtration apparatus with transmembrane pressure (TMP) set at 1 bar. A three (3) centimeter (cm) diameter membrane was cut off and installed in the membrane sample holder with an effective filtration diameter of 2.8 cm. The amount of water permeated was sampled and the weight obtained every 30 min until a stable or uniform flux was obtained for three (3) consecutive readings.

For bacterial analysis, three pieces of 10 mm diameter discs were cut off from the dried membrane and subjected for antimicrobial assay by the disc diffusion method using *E. coli* as a test organism. The analysis was done in triplicate and in addition to membrane samples each setup also included thirty (30) micrograms (μg) of amikacin as positive control and a sample-free disc for the negative control. Sample coding use by the microbiological laboratory was adopted in the presentation of results. The results were evaluated in terms of reactivity and inhibitory activity rating as described in the Supplementary Materials.

## 3. Results and Discussion

### 3.1. PTU Polymer Characterization

**Figure 1:**
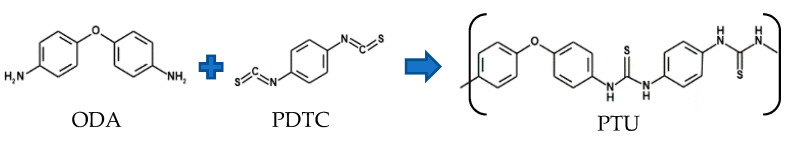
Polythiourea Synthesis Route and Chemical Structure.

In this study, PTU was formed through condensation polymerization of 4,4-oxydianiline (ODA) and p-phenylene diisothiocyanate (PDTC) in dimethyl sulfoxide (DMSO), the structure of which is shown in Figure 1. The reaction produces a viscous solution which confirms the formation of the polymer and measured average MW by the SLS technique of 451 ± 17.59 kDa (kilodalton) (see Supplementary Materials). To further validate the structure of PTU, an FTIR analysis of PTU was compared with the FTIR of reactants.

**Figure 2:**
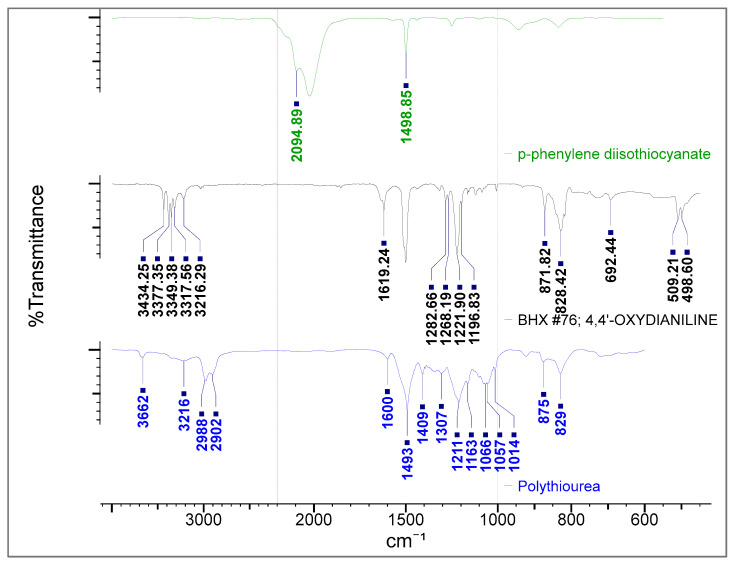
Overlay of FTIR Spectra of ODA, PDTC and PTU.

The overlay of the FTIR Spectra of PTU and of the reactants obtained from online FTIR database/library use for synthesis namely ODA [35] and PDTC [36] is presented in Figure 2. In the FTIR spectra of PDTC, the broad and strong peak at 2023 cm^−1^ attributed to isothiocyanate (S=C=N) stretching disappears completely in the spectra of PTU. The Isothiocyanate functional group totally reacted during the polymerization as depicted in its structure. The wave numbers at 1220 cm^−1^ and 1500–1493 cm^−1^, attributed to CO and aromatic C–C stretching, respectively, are present both in ODA and PTU with a slight shift in wave number [21]. The broad peak starting from 1568 cm^−1^, which overlaps with the 1500 cm^−1^, includes the C=S stretching, which is absent in both the PDTC and ODA spectra. The broad peak at the 3000–3600 cm^−1^ range is attributed to N–H stretching where the PTU spectra shifted to the left compared with the ODA spectra possibly due to its reaction to the isothiocyanate group.

### 3.2. Membrane Chemistry

The minimum concentration of Cu(OAc)_2_ used in the coagulant bath is about 5 mM which corresponds to the concentration resulting to skin layer formation in CIPS where Cu(OAc)_2_ in DMSO alone is used [19]. At this concentration, the amount of crosslinking formed instantaneously is enough to prevent the dissolution of the polymer since the same solvent is use for phase separation resulting to a flat and uniform surface. Below this concentration, some of the polymer may escape the casted dope resulting to non-uniform membrane surface and dispersion of crosslinked polymer in the solvent bath containing the cross linkers.

**Figure 3:**
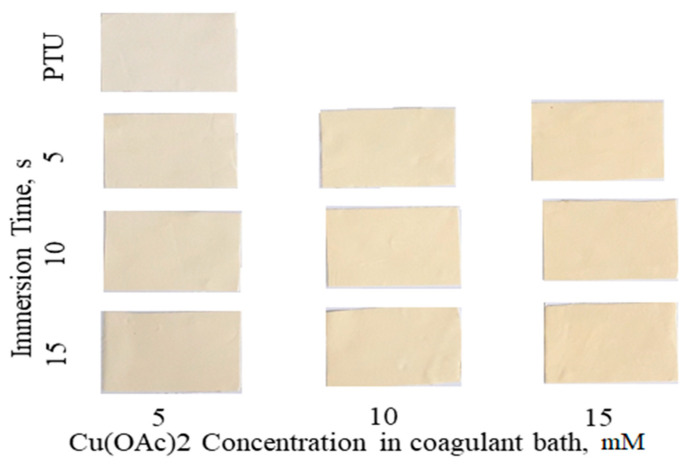
Fabricated PTU and PTU-Cu Membranes.

During immersion of the cast dope in the coagulant bath, the color changes to yellow with intensity proportional to the concentration of Cu(OAc)_2_ as shown in Figure 3. However, at constant concentration, the change in color is not as intense with longer immersion time indicating that reaction of Cu occurs immediately at the onset of phase separation through NIPS. Through NIPS the polymer precipitates and immobilized instantaneously inhibiting further the rate of reaction with Cu. The crosslinking of PTU-Cu was also verified by dissolving the membrane in DMSO and retrieving a significant amount of undissolved polymer after 5 days.

**Figure 4:**
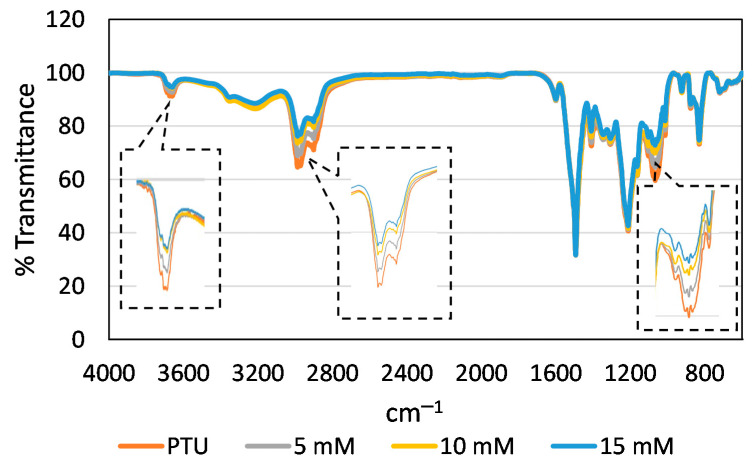
FTIR Spectra of PTU and PTU-Cu Membranes.

Overlay of the FTIR Spectra of the pristine PTU membrane and the PTU-Cu Composite Membrane also confirms reaction of Cu with the polymer as shown in Figure 4. Majority of the functional groups of the polymer are still present in the composite membrane since only the surface and a small amount of the inner layer reacted to Cu. No new peaks were evident indicating the lack of vibrational frequencies of the crosslink that forms. However, there is a decline in intensity of various peaks of PTU-Cu relative to PTU as presented in Table 1. The functional groups associated with these wavenumbers may have participated in the Complexation with Cu as depicted by decrease in intensity/activity correlates with increase in Cu concentration. The formation of metal-polymer complex decreases the intensity of the sulfur and amine containing functional groups. The results were consistent with the Cu containing ions detected by TOFSIMS namely CH_2_NCu^+^ and CuN^+2^ for the positive ion and CuNH^−2^, CNCu^−^, CHNCu^−^ and CuS^−^ for the negative ion.

**Figure 5:**
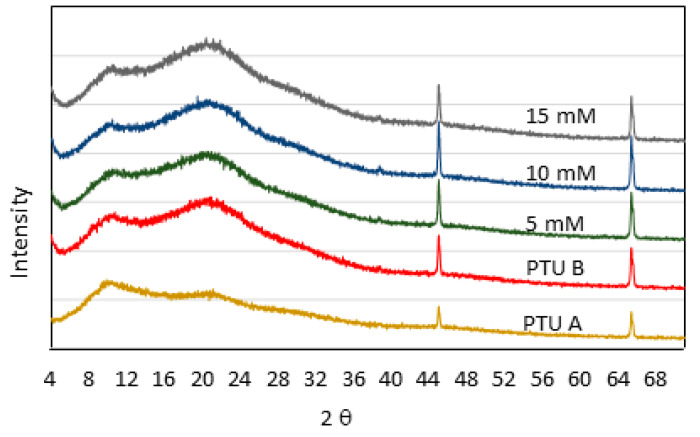
XRD Profile of PTU and PTU-Cu Membranes.

The results of XRD analysis for PTU membrane and PTU-Cu membrane immersed in coagulant bath for 10 s are shown in Figure 5. All membranes showed a similar profile with varying intensity of two peaks at 2θ for values of 43.96 and 64.32 corresponding to the increase in Cu concentration in the bath. Based on the XRD, the synthesized polymer was amorphous, making it suitable for membrane application [37]. The shift of peaks in XRD for the amorphous component of PTU that was exposed to ambient conditions may indicate possible polymorphism of PTU.

**Figure 6:**
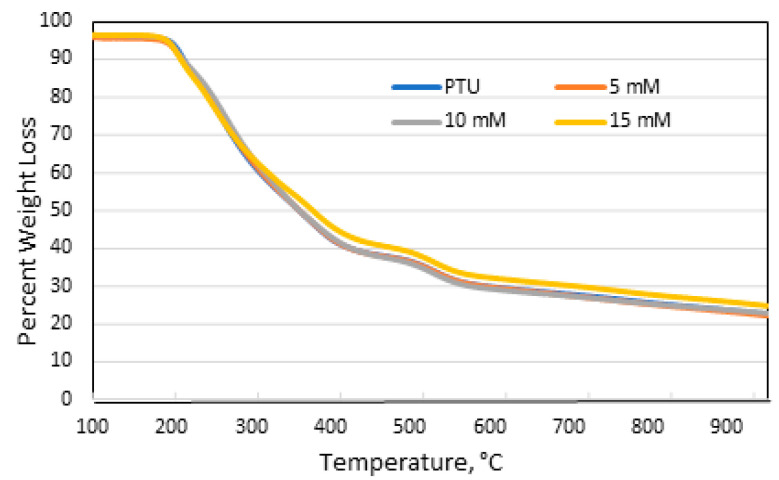
TGA Profile of PTU and PTU-Cu Membranes.

TGA analysis showed an almost identical pattern where the profile for all of the membrane overlaps were as presented in Figure 6. This is to be expected since only a small portion of the membrane complexed with Cu and the majority of the membrane was still made up of the polymer. Residual analysis data for PTU and membrane immersed in a coagulant bath containing 5, 10, and 15 mM Cu(OAc)_2_ are 22.91%, 22.59%, 23.05%, and 25.06%, respectively. The percent residual at temperatures greater than 900 °C increases with an increase in Cu concentration. The onset of degradation temperature is around 200 °C, close to the results of Ma et al. [21].

**Figure 7:**
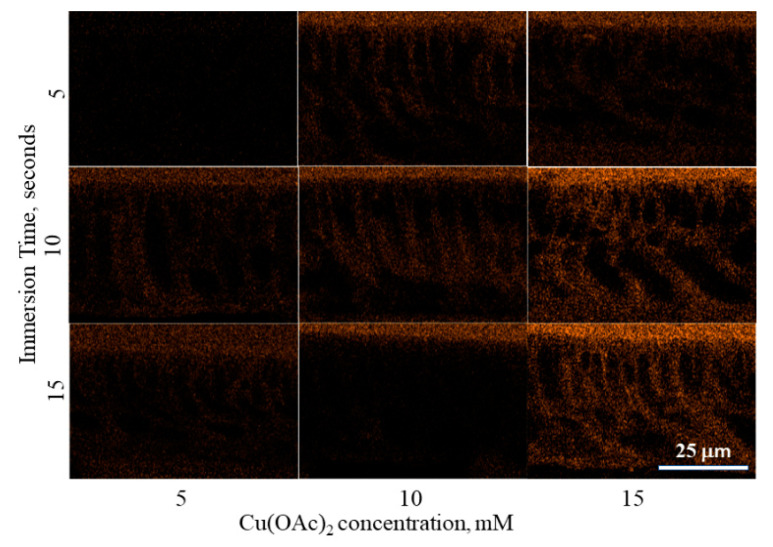
EDX Map of Cu in PTU-Cu Membrane Cross Section.

The diffusing coagulant bath containing Cu also results in crosslinking of the polymer within the membrane. EDX Mapping of Cu at the cross section of the membrane as shown in Figure 7 confirms the deposition of Cu. With an immersion time of from 5 to 15 s only, the deposition indicates a relatively fast reaction and formation of the Cu-polymer crosslink within the pore walls as the coagulant diffuses. While its concentration was higher at the surface of the membrane, a significant amount was also deposited throughout the polymer matrix. The diffusion pathway of the coagulant is also depicted, which starts at the surface, changing to less intense color within the membrane.

As the coagulant diffuses throughout the membrane, the Cu concentration is depleted. At low concentration of Cu(OAc)_2_ in the coagulant bath and short immersion time, the presence of copper within the membrane is relatively low. This is due to the reaction of Cu with the polymer decreasing its concentration in the distilled water.

Given the mechanism of the process, pore former additives such as polyethylene glycol (PEG) and polyvinyl pyrollidone (PVP) may have different effects on the deposition of Cu due to different distributions of PTU within the dope.

### 3.3. Membrane Morphology

**Figure 8:**
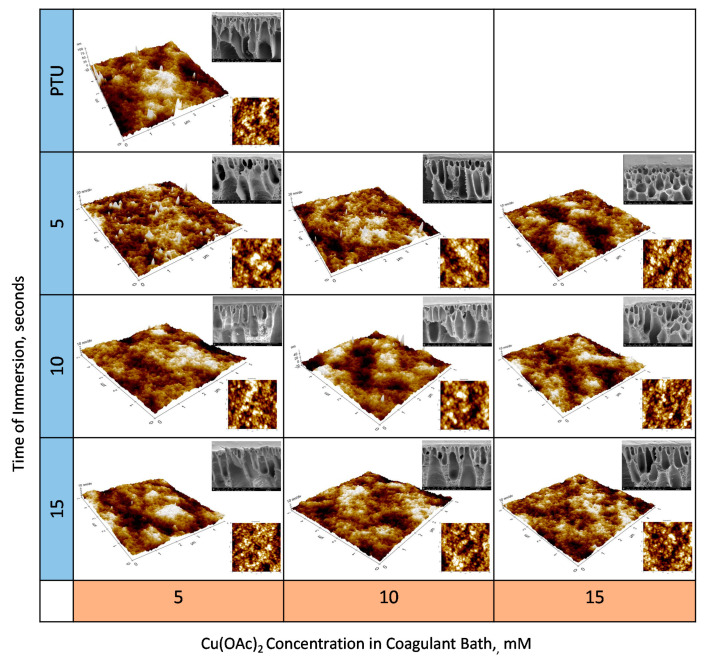
AFM and SEM Images of the PTU-Cu Membranes.

The effects of the Cu on the membrane are manifested at the surface. Three-dimensional AFM images of the surface morphology of the membrane surface are shown in Figure 8 with SEM images of the membrane cross section at 10,000× magnification. Generally, results show that the membrane with Cu had a smoother surface compared with the pristine PTU membrane. In addition, the insets of AFM images with scan size of 500 nm × 500 nm show polymer nodules and surface pores of the membranes in nanofiltration range. This is typical for a membrane fabricated through NIPS.

SEM images of the cross section of the membrane show dense skin layer. All of the fabricated membrane also shows a finger-like macrovoid structure due to a fast solvent–nonsolvent exchange [38] in the DMSO–water system.

It can also be seen from the SEM images that the point of breakage resulting from the preparation of membrane samples through cryo-snap shows some extended projection for membrane containing Cu. This indicates some degree of flexibility of the material at lower temperature relative to the pristine PTU membrane possibly due to crosslinking within the membrane pore walls.

### 3.4. Effect of Time of Immersion and Concentration

The simultaneous phase separation process use in membrane fabrication did not have noticeable effects visually on the membrane’s morphology at the surface and cross section. However, it had an effect on the properties and performance of the membrane. Both the time of immersion and concentration of Cu in the coagulant bath had significant influence on the overall performance of the membrane.

**Figure 9:**
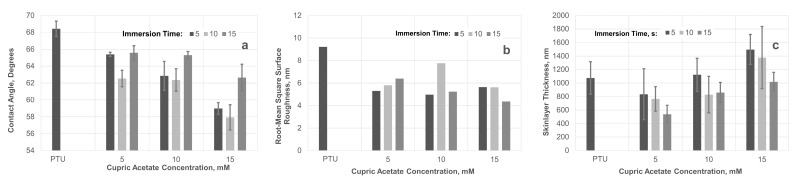
Effect of Time and Immersion on (**a**) Contact Angle, (**b**) Root Mean Square Roughness, and (**c**) Skin Layer Thickness.

Changes in surface chemistry and morphology brought about by varying these fabrication parameters influenced the surface energy of the membrane. This could be measured using the water contact angle, which indicates the degree of hydrophilicity of the membrane’s surface. The contact angle of the PTU-Cu membrane decreased as the concentration of Cu(OAc)_2_ increased as shown in Figure 9a. On the other hand, the contact angle decreased only up to 10 s of immersion time and increased at 15 s, but still below that of the pristine PTU membrane. Phase separation by NIPS and CIPS occurred upon contact of the dope solution with the coagulant. Further exposure of the nascent membrane with Cu(OAc)_2_ after 10 s may result in saturation of the surface with Cu. This results in changes in surface energy and morphology which consequently affects the contact angle [39]. In comparison, the surface of the membrane fabricated through CIPS only was relatively hydrophobic [40]. The use of water in the complexation reaction during membrane formation may have an effect on the membrane surface characteristics due to the different nature of the solvent/coagulant used during skin layer formation. Fouling formation, in particular biofouling, was also inhibited [41] with membranes that are more hydrophilic. Due to the hydrophobic nature of the foulant, it was easily washed off by shear forces in a cross-filtration setup. Similarly, the surface roughness of the PTU-Cu decreased with the incorporation of Cu, indicating a smoother membrane relative to the pristine PTU membrane, as presented in Figure 9b. This may be attributed to intra- and inter-crosslinking of polymer and bridging interstitial spaces within and across polymer molecule. A smoother membrane tended to foul less often due to less surface area to which foulants can attach [42,43]. However, variation of surface roughness with time of immersion in coagulant bath and concentration of Cu(OAc)_2_ varied probably due to interruption of solvent exchange and movement brought about by transfer of the nascent membrane to the second bath. At 15 s immersion time, a decreasing trend in surface roughness was observed with an increase in the concentration of Cu(OAc)_2_ in the first bath. This duration of immersion at the coagulant bath may have stabilized the membrane in terms of structure and morphology.

In NIPS, pores were formed at the surface through the diffusion of solvent into the non-solvent bath and vice versa [44]. In the same manner, simultaneous CIPS and NIPS also formed surface pores due to diffusion of solvent and non-solvent at the polymer–coagulant bath interface. Therefore, the observed decrease in surface roughness and hydrophilization of the membrane surface could extend within the pore walls.

The thickness of the skin layer indicates some dependency on Cu(OAc)_2_ concentration and immersion time as shown in Figure 9c. An increase in the skin layer thickness was associated with an increase in Cu(OAc)_2_ concentration, while a reverse trend was observed with an increase in immersion time. A high concentration of Cu(OAc)_2_ resulted in more instances of phase separation at the interface. On the other hand, further exposure of the nascent membrane with Cu(OAc)_2_ resulted to shrinkage of the skin layer.

### 3.5. Membrane Performance

**Figure 10:**
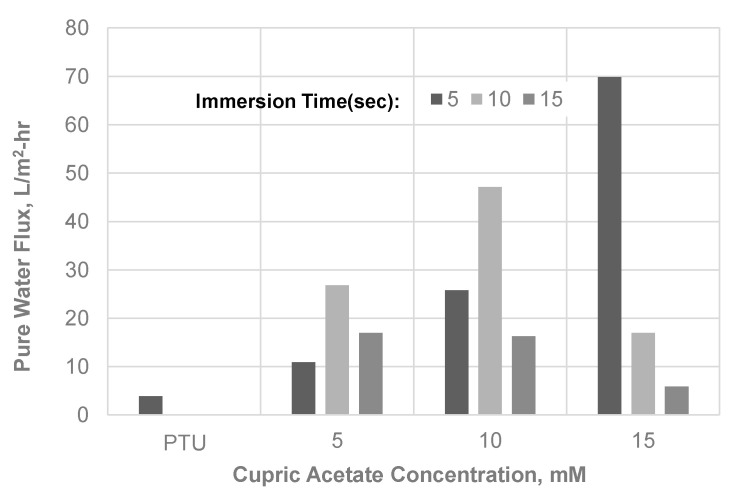
Pure Water Flux of PTU and PTU-Cu Membranes.

Membrane Performance was evaluated in terms of pure water flux and antibacterial property. The pure water flux of the membrane with Cu was relatively higher compared with the pristine PTU membrane, as shown in Figure 10. The increase in hydrophilicity and the decrease in membrane surface roughness of the membrane may be a factor in the increase in water flux. At 5 s immersion time, an increase in water flux was observed with an increase in Cu(OAc)_2_ concentration in the coagulant bath. Water flux with immersion time of 10 s or more showed varying water flux with concentration probably due to the formation of Cu moieties at the pores. In addition, the observed increase in thickness of the skin layer with increase in time of immersion and concentration may also have contributed to the observed variation in water flux. In comparison, several studies on physically doped metal on membrane, particularly in its nanoparticle form, showed the same [6,45] or a decrease [46,47] in flux due to blockage of the pores or reduction in the free volume brought about by the presence of nanoparticle. In contrast, metal oxide nanoparticle such as nanotitania (TiO_2_) [48] or nano-copper oxide (CuO) [8] showed an increase in flux mainly attributed to the increase in hydrophilicity of the surface of the membrane. In addition, the chemical association of metal such as silver as studied by Prince et al. [49] resulted in an increase in flux by up to 39.4% and a decrease in the contact angle of 14–48%.

**Figure 11:**
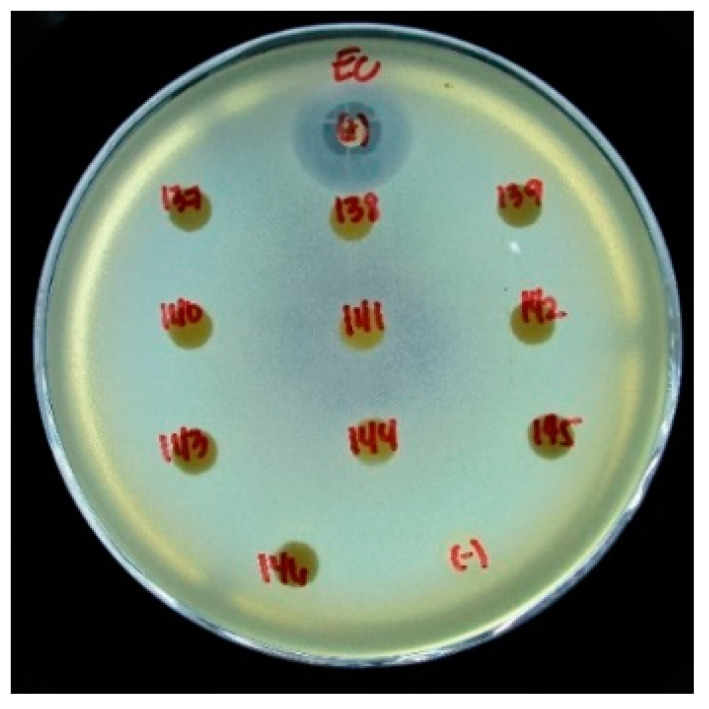
Antibacterial Test Results of the PTU and PTU-Cu Membranes.

All the membranes fabricated exhibited slight to partial antibacterial activity limited under the membrane surface as shown in Figure 11 and described in Table 2 where the membrane disc samples are visible. In comparison, the negative control disc is not visible due to growth of *E. coli* underneath. Since the zone of inhibition did not extend outside the membrane, this indicates no diffusion of antibacterial materials. Other studies that physically incorporate nano-Cu or nano-Ag in the membrane observed an extended zone of inhibition beyond the membrane material. However, this resulted in leaching of the metal, thereby reducing its antifouling efficacy in membrane filtration applications [6,46]. This problem can be addressed with chemical association based on the study of Prince et al. [49]. The presence of Cu in the membrane was shown to inhibit bacterial attachment and biofilm formation [9]. This antibacterial property has the potential to impart an anti-fouling property to the membrane with extended lifetime due to the non-diffusion of the active material. Further testing using other microorganisms and actual evaluation of antifouling properties needs to be conducted to validate the results.

Interestingly, at a Cu(OAc)_2_ concentration of 10 mM, the inhibitory activity rating was only slight relative to other concentrations. At this concentration, the Cu may have saturated the reaction sites of the polymer and it is proposed that these sites are also responsible for the antibacterial characteristics of the polymer. At a higher concentration, the antibacterial property may be attributed to Cu, while at a low concentration it is attributed to the polymer. With simultaneous physical and chemical phase separation, the effect of immersion time on the inhibitory rating of the surface is not significant due to the instantaneous immobilization of the polymer by both phase separation processes.

## 4. Conclusions

A PTU-Cu composite membrane was successfully fabricated via simultaneous NIPS and CIPS to strategically incorporate copper in the membrane. This technique provided a new perspective in membrane fabrication where two instantaneous distinct physical and chemical phase separation processes were incorporated in a single step. Cross sections of all membranes have similar morphology as exhibited by dense skin layer and finger-like support due to high miscibility of DMSO and water.

FTIR spectra show a proportionate decrease in sulfur and amine functional group intensity with increasing concentration of Cu in the coagulant bath, confirming their interaction with Cu. TOFSIMS results also confirm these Cu moieties at the surface of the membrane.

The diffusion of the coagulant into the dope solution provided strategic pore enhancement through crosslinking. Its synergistic effect on the characteristics and performance of the membrane was also evident. Compared to the pristine PTU membrane, the presence of Cu results in a reduction in the contact angle and surface roughness, which may also extend to the pores of the membrane. In effect, this increases the pure water flux as a result of incorporation of Cu. The skin layer of the membrane increases in thickness with an increase in concentration of Cu(OAc)_2_ in the coagulant bath.

Lastly, all membranes produced, including the pristine PTU membrane, show some antibacterial characteristics concerning the surface against *E. coli*.

## Figures and Tables

**Table 1:** Effect of Cu on Vibrational Frequencies of Various Functional Groups.

Wave Number, cm^−1^	Functional Groups	Effect in Intensity with Increasing Cu
~3200	N–H stretching	Decrease
~3000	Ar C–H stretching	Decrease
2900–3000	C–H Symmetric Stretching	Decrease
~1340	C–N stretching	Decrease
1240	C=S stretching	Slight decrease

**Table 2:** Inhibitory Rating of the PTU and PTU-Cu Membranes.

Membrane Code	Immersion Time (s)	Cu conc (mM)	Inhibitory Activity Rating *
137	0 (PTU)	0	++
138	5	5	++
139		10	+
140		15	++
141	10	5	++
142		10	+
143		15	++
144	15	5	++
145		10	+
146		15	+

* (++) partial; (+) slight.

## Data Availability

Not applicable.

## Supplementary Materials

The following are available online at https://www.mdpi.com/article/10.3390/polym13111743/s1, TOFSIMS Spectra of PTU-Cu Membrane.
