# Supplementary material for: Characteristics and Performance of PTU-Cu Composite Membrane Fabricated through Simultaneous Complexation and Non-Solvent Induced Phase Separation
Source: Polymers (Basel). 2021 May 26;13(11):1743. doi: 10.3390/polym13111743 (PMC8198067; doi:10.3390/polym13111743)
Supplement: Supplementary file 1 [file polymers-13-01743-s001.zip › polymers-1224823-supplementary.pdf]

2021.05.19 16:29:44

HORIBA Scientific

HORIBA SZ-100 for Windows [Z Type] Ver2.40

SZ-100

Graph Type	ID#	File Name	Measurement Type	Date
	202105171732002	PTU_2149.nml	Molecular Weight(Debye Plot)	Monday, 17 May 2021 5:32:28 PM

PTU
Replicate 1

PTU_2149.nml
Measurement Results

Date	:	Monday, 17 May 2021 5:32:28 PM
Measurement Type	:	Molecular Weight(Debye Plot)
Sample Name	:	PTU
Scattering Angle	:	90
Temperature of the Holder	:	25.0 °C
Plot Type	:	Debye
Concentration	:	2.000, 4.000, 6.000, 8.000 mg/mL
Rayleigh Ratio	:	6.100 x 10^{-6} cm^{-1}
Refractive Index Increment	:	0.072 mL/g
Standard Dispersant Name	:	DMSO-Std
Refractive Index of Standard Dispersant	:	1.477

Calculation Results

Molecular Weight	:	432.2 kDa
Second Virial Coefficient	:	1.549 x 10^{-4} mL mol/g^2
P^{-1}(Theta)	:	1.000

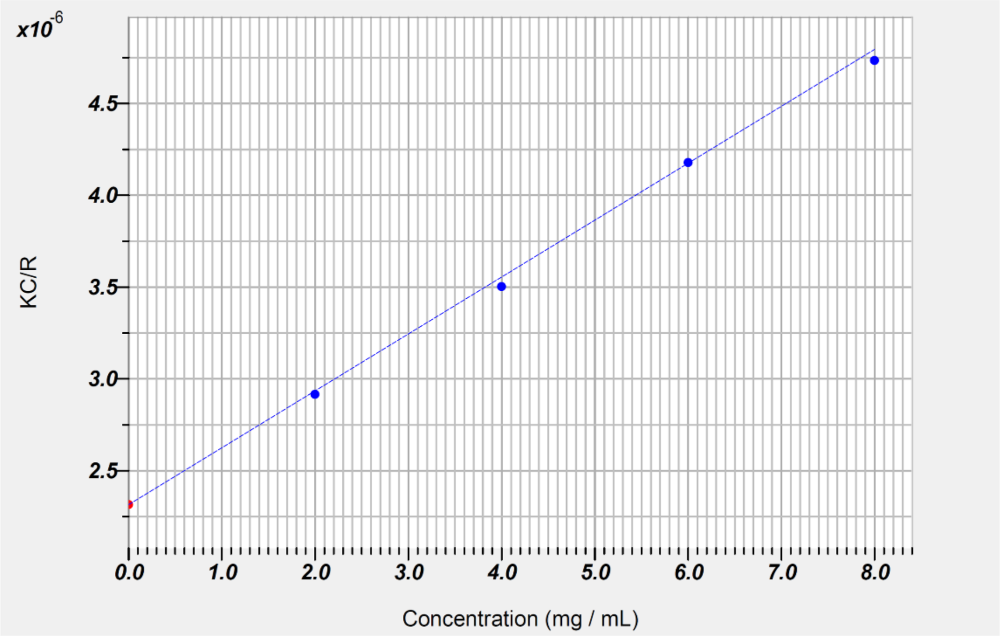

HORIBA Scientific

HORIBA SZ-100 for Windows [Z Type] Ver2.40

SZ-100

Graph Type	ID#	File Name	Measurement Type	Date
	202105181710011	PTU-Q2_2162.nml	Molecular Weight(Debye Plot)	Tuesday, 18 May 2021 5:10:21 PM

PTU
Replicate 2

PTU-Q2_2162.nml
Measurement Results

Date	:	Tuesday, 18 May 2021 5:10:21 PM
Measurement Type	:	Molecular Weight(Debye Plot)
Sample Name	:	PTU-Q2
Scattering Angle	:	90
Temperature of the Holder	:	24.8 °C
Plot Type	:	Debye
Concentration	:	2.000, 4.000, 6.000, 8.000 mg/mL
Rayleigh Ratio	:	6.100 x 10^{-6} cm^{-1}
Refractive Index Increment	:	0.072 mL/g
Standard Dispersant Name	:	DMSO-Std
Refractive Index of Standard Dispersant	:	1.477

Calculation Results

Molecular Weight	:	466.6 kDa
Second Virial Coefficient	:	4.601 x 10^{-5} mL mol/g^2
P^{-1}(Theta)	:	1.000

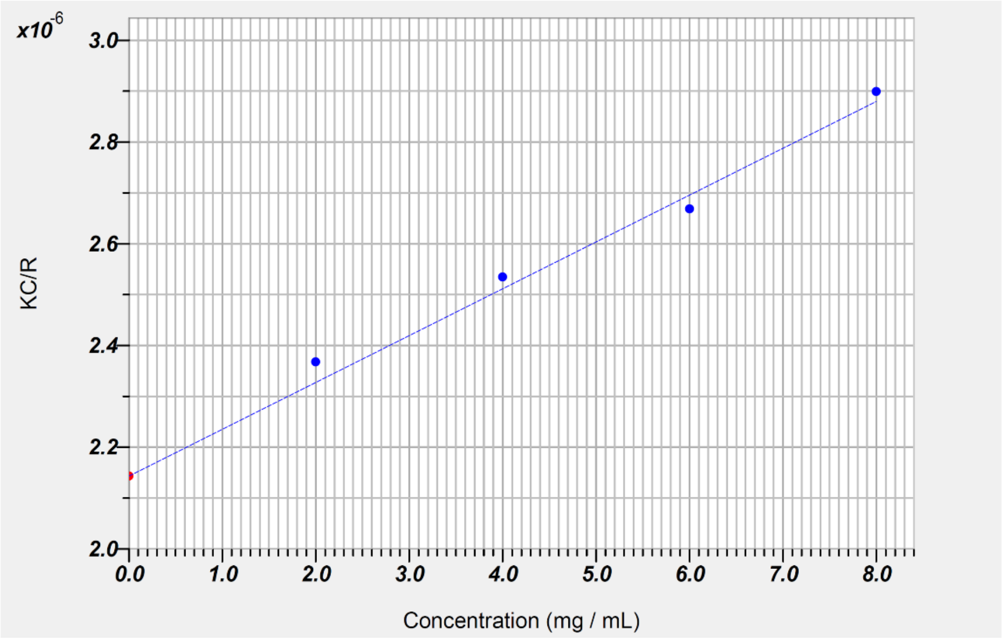

HORIBA Scientific

HORIBA SZ-100 for Windows [Z Type] Ver2.40

SZ-100

Graph Type	ID#	File Name	Measurement Type	Date
	202105182015012	PTU-Q2.1_2163.nml	Molecular Weight(Debye Plot)	Tuesday, 18 May 2021 8:15:37 PM

PTU
Replicate 3

PTU-Q2.1_2163.nml
Measurement Results

Date	:	Tuesday, 18 May 2021 8:15:37 PM
Measurement Type	:	Molecular Weight(Debye Plot)
Sample Name	:	PTU-Q2.1
Scattering Angle	:	90
Temperature of the Holder	:	25.0 °C
Plot Type	:	Debye
Concentration	:	2.000, 4.000, 6.000, 8.000 mg/mL
Rayleigh Ratio	:	6.100 x 10^{-6} cm^{-1}
Refractive Index Increment	:	0.072 mL/g
Standard Dispersant Name	:	DMSO-Std
Refractive Index of Standard Dispersant	:	1.477

Calculation Results

Molecular Weight	:	455.8 kDa
Second Virial Coefficient	:	3.710 x 10^{-5} mL mol/g^2
P^{-1}(Theta)	:	1.000

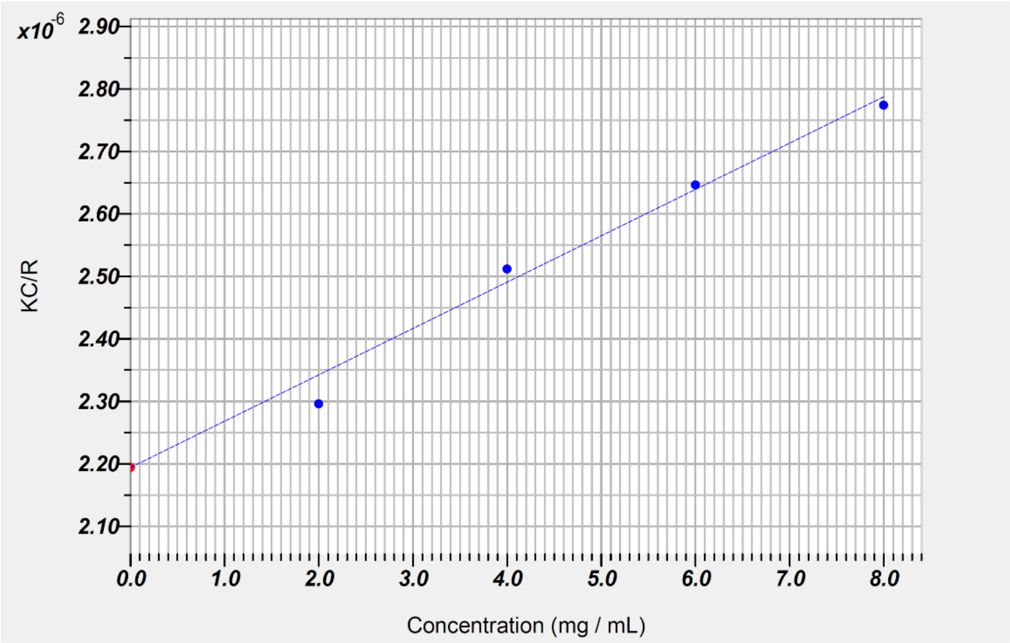

REPUBLIC OF THE PHILIPPINES
DEPARTMENT OF SCIENCE AND TECHNOLOGY
INDUSTRIAL TECHNOLOGY DEVELOPMENT INSTITUTE
STANDARDS AND TESTING DIVISION

ORIGINAL RECEIVED
BY: [illegible]
DATE: 3/22/2021

TEST REPORT

ITDI-022021-MIC-0056

Customer's Name	:	MSD-ITDI
Address	:	MSD Bldg., ITDI, DOST Compound, Bicutan, Taguig City
Contact Details	:	Landline No.: 8837.2071
Date Received	:	February 10, 2021
Sample	:	*Discs*
Description	:	*0137-0146) Membrane Samples Polythiourea (PTU) with Copper Label 4 pcs disc; Date Packaged: Feb. 9, 2021; 9:00 PM*
Identification	:	Submitted as *0137) PTU*<br>*0138) PTUCu55*<br>*0139) PTUCu510*<br>*0140) PTUCu515*<br>*0141) PTUCu105*<br>*0142) PTUCu1010*<br>*0143) PTUCu1015*<br>*0144) PTUCu155*<br>*0145) PTUCu1510*<br>*0146) PTUCu1515*
Date(s) Tested	:	February 18 - 19, 2021

The samples, *PTU, PTUCu55, PTUCu515, PTUCu105, PTUCu1015,* and *PTUCu155,* produced partial inhibitory activity (++) with mild reactivity (2) against the test organism, *Escherichia coli.*

The samples, *PTUCu510, PTUCu1010, PTUCu1510,* and *PTUCu1515,* produced slight inhibitory activity (+) with mild reactivity (2) against the test organism, *Escherichia coli.*

Amikacin 30 ug, which served as positive control, produced complete inhibitory activity (+++) with moderate reactivity (3) against the test organism, *Escherichia coli.*

The sample-free disc, which served as negative control, had no inhibitory activity (-) and no reactivity (0) against the test organism.

STD Building, DOST Compound, Gen. Santos Ave., Bicutan, Taguig City, Metro Manila 1631, Philippines
Tel. Nos. : **(632) 837-2071** to **82** loc **2188, 2189** (Receiving and Releasing Unit) / **2198** (Division Chief);
Telefax Nos. : **(632) 837-0032**
Email: std@itdi.dost.gov.ph; Website: http://itdi.dost.gov.ph

Sample/ Control	*Escherichia coli (ATCC 25922)*					
	Replicate 1	Replicate 2	Replicate 3	Total Mean Zone of Inhibition (mm)	Reactivity	Inhibitory Activity
PTU (10 mm)	10.00	10.00	10.00	10.00	2	++
PTU Cu55 (10 mm)	10.00	10.00	10.00	10.00	2	++
PTU Cu510 (10 mm)	10.00	10.00	10.00	10.00	2	+
PTU Cu515 (10 mm)	10.00	10.00	10.00	10.00	2	++
PTU Cu105 (10 mm)	10.00	10.00	10.00	10.00	2	++
PTU Cu1010 (10 mm)	10.00	10.00	10.00	10.00	2	+
PTU Cu1015 (10 mm)	10.00	10.00	10.00	10.00	2	++
PTU Cu155 (10 mm)	10.00	10.00	10.00	10.00	2	++
PTU Cu1510 (10 mm)	10.00	10.00	10.00	10.00	2	+
PTU Cu1515 (10 mm)	10.00	10.00	10.00	10.00	2	+
Positive Control: *Amikacin 30 ug* (6 mm)	15.05	15.03	15.11	15.06	3	+++
Negative Control: Sample-free disc (10 mm)	0.00	0.00	0.00	0.00	0	(-)

Reactivity Rating: 0 – None (No detectable zone around or under specimen)
1 – Slight (Some malformed or degenerated cells under the specimen)
2 – Mild (zone limited under the specimen)
3 – Moderate (zone extends 5 to 10 mm beyond specimen)
4 – Severe (zone extends greater than 10 mm beyond specimen)

Inhibitory Activity Rating: (+++) complete; (++) partial; (+) slight, and (-) negative

Test Reference: United States Pharmacopoeia 30-NF 25, 2007 <87> Biological Reactivity Tests, In vitro
Test Method: Disc Diffusion Method

ITDI-022021-MIC-0056
February 24, 2021

h

REPUBLIC OF THE PHILIPPINES
DEPARTMENT OF SCIENCE AND TECHNOLOGY
INDUSTRIAL TECHNOLOGY DEVELOPMENT INSTITUTE
STANDARDS AND TESTING DIVISION

VALIDITY OF THE TEST REPORT: The test results are those obtained at the time of the test and pertain only to the sample(s) received by the Laboratory of this Institute. *Codes and words in Italics are quoted solely for the customer's reference; significance of these codes and words is not verified by the Laboratory.* This report is not to be used for advertising purposes or sales promotion. This report shall not be reproduced except in full without the approval of the Standards and Testing Division.

GP 5.10-01-F06
Rev 1 / 28 June 2018

2/24/21

MARLON SA. AGUINALDO, RMT
Head/Technical Manager, Microbiology Section

DR. CYNTHIA M. NALO-OCHONA
Head, Over-all Technical Manager
Biological Laboratory

Issued under the Authority of:

ROSALINDA C. TORRES, PhD, RCh
Division Chief/Quality Manager

Quality Life and Products Through Testing.

STD Building, DOST Compound, Gen. Santos Ave., Bicutan, Taguig City, Metro Manila 1631, Philippines
Tel. Nos. : **(632) 837-2071** to **82** loc **2188, 2189** (Receiving and Releasing Unit) / **2198** (Division Chief);
Telefax Nos. : **(632) 837-0032**
Email: std@itdi.dost.gov.ph; Website: http://itdi.dost.gov.ph

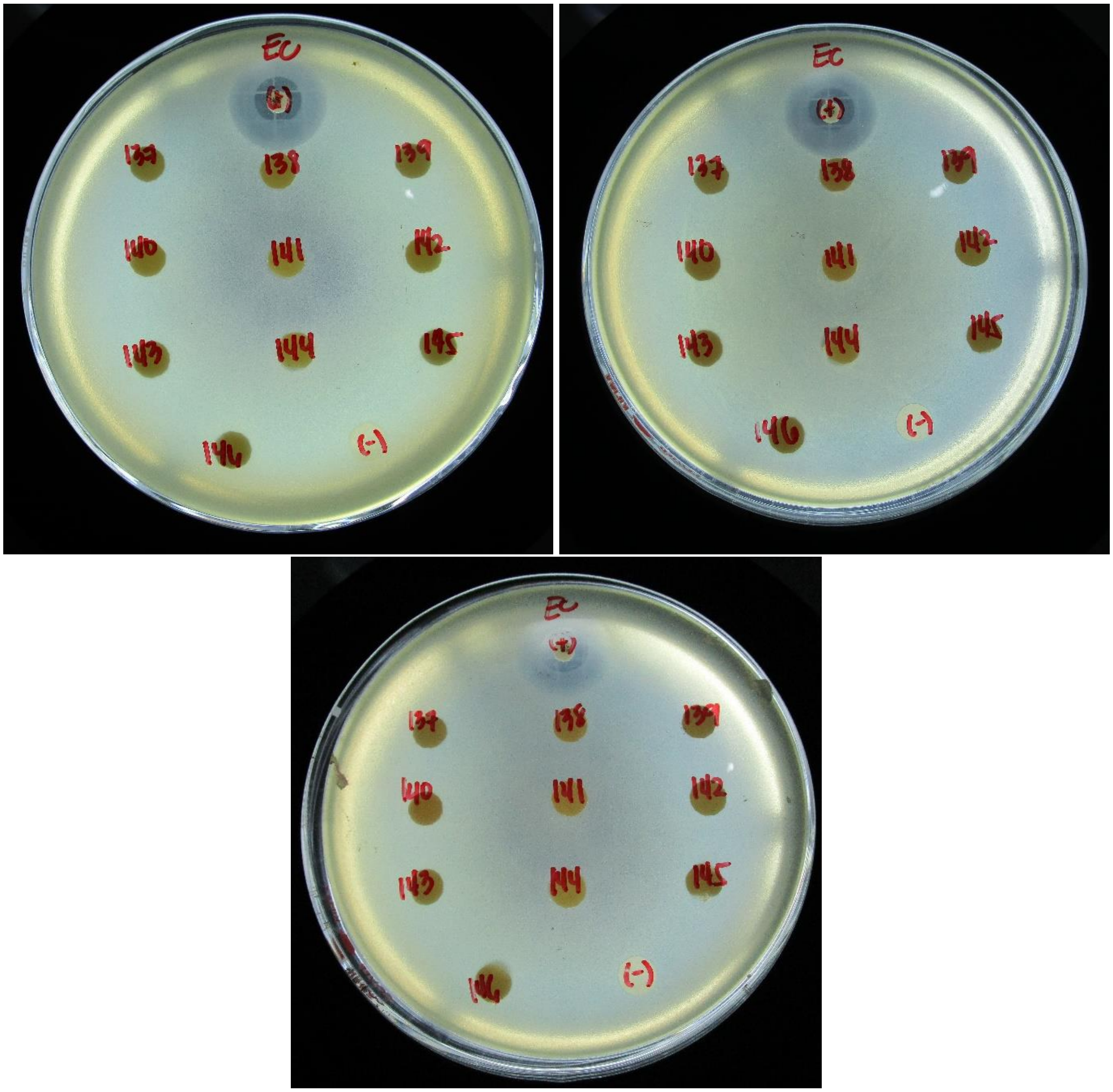

Replicate

TOFSIMS Spectra of PTU-Cu Membrane

Positive Ion

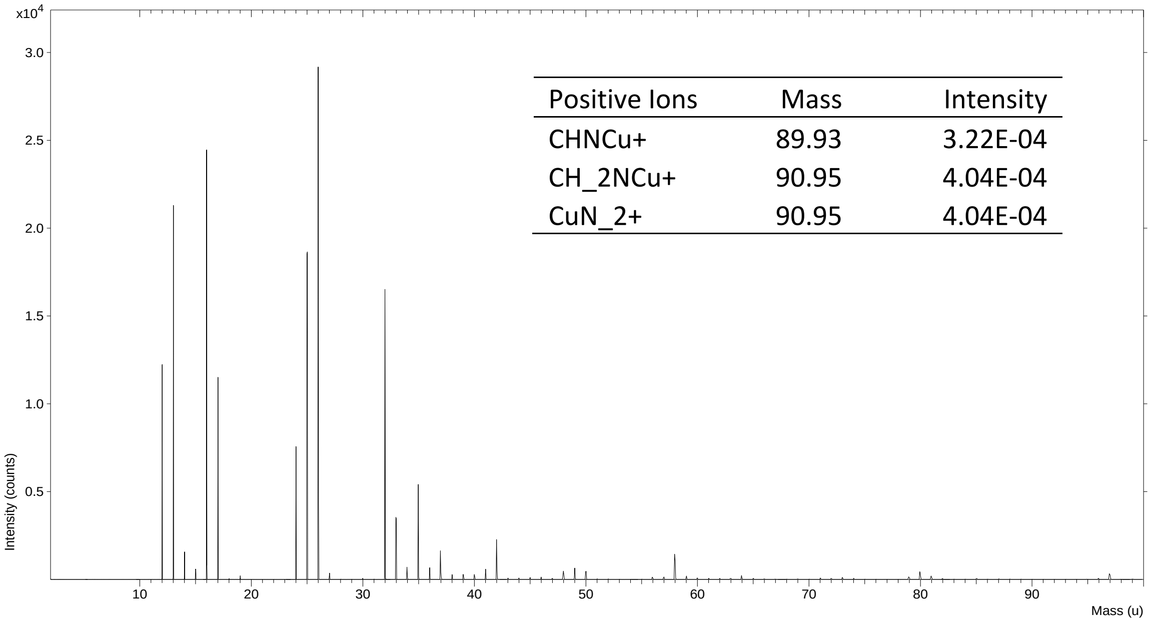

Positive Ions	Mass	Intensity
CHNCu+	89.93	3.22E-04
CH_2NCu+	90.95	4.04E-04
CuN_2+	90.95	4.04E-04

Negative Ion Spectra

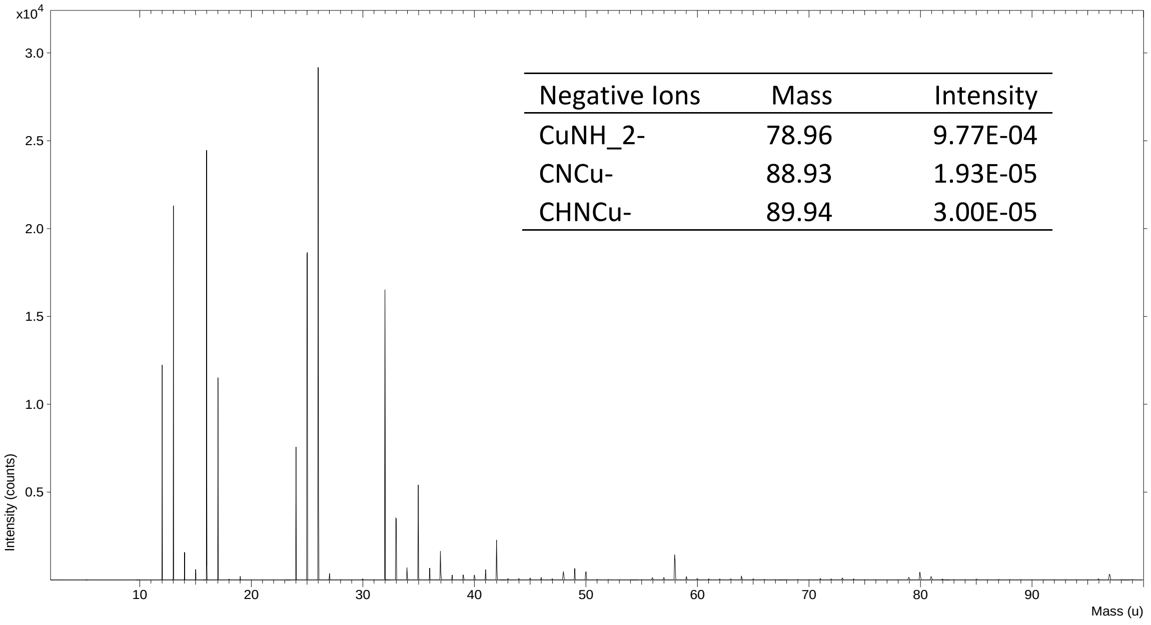

Negative Ions	Mass	Intensity
CuNH_2-	78.96	9.77E-04
CNCu-	88.93	1.93E-05
CHNCu-	89.94	3.00E-05
